# The chemosensory systems of *Vibrio cholerae*

**DOI:** 10.1111/mmi.14520

**Published:** 2020-05-13

**Authors:** Davi R. Ortega, Andreas Kjær, Ariane Briegel

**Affiliations:** ^1^ Institute of Biology Leiden University Leiden The Netherlands; ^2^ Department of Biochemistry University of Oxford Oxford UK; ^3^ Present address: Division of Biology and Biological Engineering California Institute of Technology Pasadena CA USA

**Keywords:** chemoreceptors, chemotaxis, *Vibrio cholerae*

## Abstract

*Vibrio cholerae*, the causative agent of the acute diarrheal disease cholera, is able to thrive in diverse habitats such as natural water bodies and inside human hosts. To ensure their survival, these bacteria rely on chemosensory pathways to sense and respond to changing environmental conditions. These pathways constitute a highly sophisticated cellular control system in Bacteria and Archaea. Reflecting the complex life cycle of *V. cholerae*, this organism has three different chemosensory pathways that together contain over 50 proteins expressed under different environmental conditions. Only one of them is known to control motility, while the function of the other two remains to be discovered. Here, we provide an overview of the chemosensory systems in *V. cholerae* and the advances toward understanding their structure and function.

## Graphical abstract

The bacterium causing cholera disease, *V. cholerae*, has three chemosensory pathways. The multiple proteins of these systems are involved in the control of cellular functions at different stages of its complex life cycle. Currently, we know that one of these systems controls motility, while the role of the other two remains unknown. Here, we provide an overview of the latest research advances aimed toward understanding the chemosensory systems in *V. cholerae*.

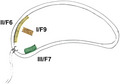

## 1 INTRODUCTION

The human pathogen *Vibrio cholerae* can thrive in a wide variety of vastly different environments: in salt‐, brackish‐, or fresh water, as free‐living cells or in biofilms on zooplankton, phytoplankton or abiotic surfaces, or as a pathogen in a host organism (Teschler *et al*., 2015). A chemosensory system allows these motile bacteria to adjust to their surroundings and finding suitable ecological niches during their planktonic lifestyle. In general, chemotactic bacteria that frequently need to adapt to changing environments tend to have more receptor genes encoded in their genome (Bardy *et al*., 2017). Therefore, it is no wonder that the chemosensory system in *V. cholerae* is exquisitely complex: It has 43 chemoreceptors distributed over both of the organisms' two chromosomes, and the core chemosensory genes are clustered in three gene clusters. Despite this complicated system, a growing number of studies have begun to shed light on structure and function of the chemosensory systems in *V. cholerae*.

## 2 THE PARADIGM: THE CHEMOSENSORY SYSTEM IN THE MODEL ORGANISM *Escherichia Coli*

Chemosensory is best understood as the chemotaxis pathway in the model bacterium *E coli* (Parkinson *et al*., 2015), Figure1. This organism has a single chemosensory system that senses the binding state of attractants and repellents to four transmembrane receptors called methyl‐accepting chemotaxis proteins (MCPs). These receptors detect nutrients, signaling molecules and toxins that bind to their periplasmic domains either directly or indirectly (Milburn *et al*., 1991; Tam and Saier, 1993; Englert *et al*., 2010). An additional fifth receptor is a methylation‐independent redox sensor that functions via the regulation of oxidation and reduction of a flavin adenine dinucleotide moiety that binds to a periplasmic period circadian protein, aryl hydrocarbon receptor nuclear translator protein, single‐minded protein (PAS) domain (Bibikov *et al*., 2004).

**FIGURE 1:**
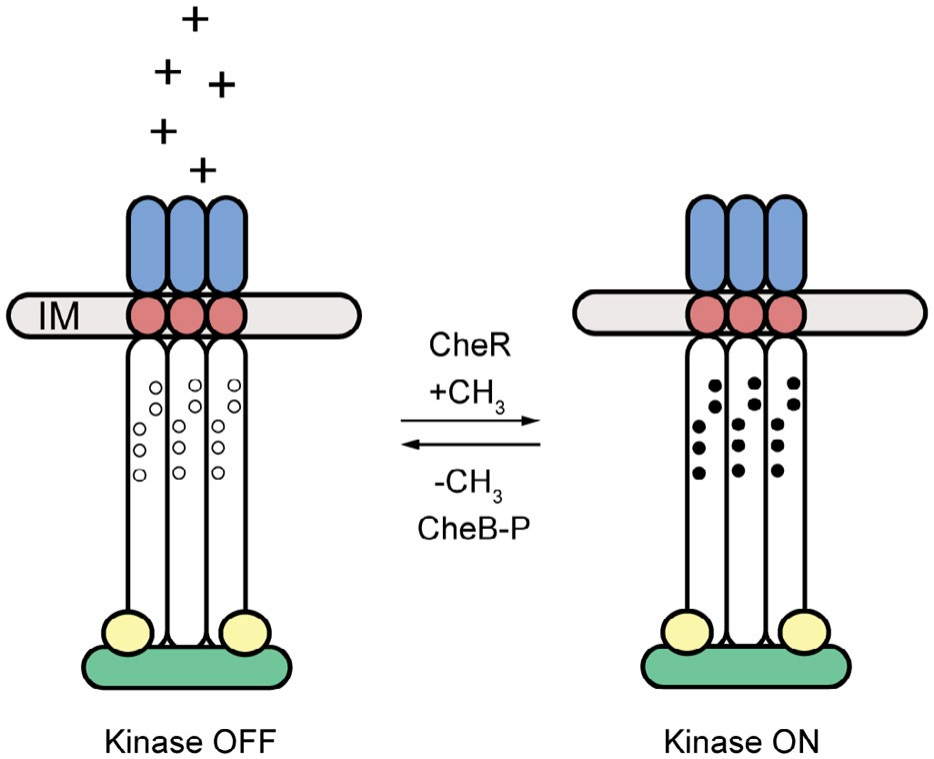
Schematic of the chemosensory signaling mechanism paradigm in *E. coli*. Here, the chemoreceptors form a membrane‐bound complex together with the histidine kinase CheA (green) and the scaffold protein CheW (yellow). Upon increase in ligand concentration (left), the periplasmic domain (blue) of chemoreceptors transduces signal through the transmembrane region (red), and the cytoplasmic domains (white) of the chemoreceptors, composed of histidine kinases, adenylate cyclases, methyl accepting proteins and phosphatases and the signaling domain. The chemoreceptors turn kinase activity off in the presence of an attractant. In the absence of attractants or presence of repellents (right), the chemoreceptors activate the kinase. The methyltransferase CheR and methylesterase CheB control the methylation state of the receptors in response to CheA activity, thereby providing a feed‐back adaptation mechanism

The binding state of the receptors is communicated through a histidine kinases, adenylate cyclases, methyl accepting proteins and phosphatases (HAMP) domain to their cytoplasmic tips. Here, they regulate the autophosphorylation of the histidine kinase CheA. This kinase is a five‐domain enzyme that forms homodimers. Each domain plays a distinct role in the function of CheA: P1 carries the substrate histidine that receives the phosphoryl group during autophosphorylation, P2 is the binding site for the response regulator CheY. Both, the P1 and P2 domains are attached to the rest of the protein via flexible linkers. P3 is the dimerization domain, P4 is the kinase domain with the ATP binding pocket, and P5 is the receptor‐binding domain (Muok *et al*., 2020).

Upon activation, the kinase transfers the phosphoryl group to CheY. Phosphorylated CheY in turn binds to the flagellar motor where it causes a switch in the direction of flagellar rotation (Stock and Da Re, 2000). When the levels of attractants are increasing, the kinase is turned off and CheY is not phosphorylated. In the absence of CheY‐P, the motors spin counter‐clockwise (ccw). This causes a bundling of the multiple flagella that together propel the cell forward, resulting in a smooth swimming pattern (a so called “run”). Upon the decrease of attractants or increase of repellents, the kinase is activated by the receptors. CheY is being phosphorylated, resulting in a reversal of motor spinning direction upon CheY‐P binding. This causes the flagellar bundle to separate and the cells randomly reorient themselves (they “tumble”). In order to keep the overall CheY‐P level in synchrony with CheA activity, CheY‐P is rapidly dephosphorylated by the phosphatase CheZ (Parkinson *et al*., 2015).

The chemotaxis system can adapt to current conditions via the regulation of receptor methylation at specific glutamyl residues in the cytoplasmic portion of the MCPs (Sourjik and Wingreen, 2012). The methylesterase CheB is also activated by CheA phosphorylation. Together with the constitutively active methyltransferase CheR, these enzymes control the methylation of the receptors which allows precise adaptation by countering the activation state of the receptors in a time delayed fashion: A fully methylated receptor results in high‐kinase activity, while a fully demethylated receptor downregulates the kinase (Parkinson *et al*., 2015). The chemotaxis system allows the cells to control the duration and frequency of run and tumble phases. Ultimately, by changing the balance between these two states, the cells move in a “biased random walk” up attractant gradients and down repellent gradients.

The receptors together form large clusters at the cell poles. In *E. coli*, receptors form well‐ordered hexagonal arrays with CheA and CheW; CheW and the CheW‐homologous P5 domain of CheA form alternating rings bound to the receptor tips. The P3 dimerization domains of CheA form a bridge between neighboring hexagons. The stoichiometry of CheA to CheW ranges from 1:1 to 1:2. This variability of CheW originates from the architecture of the array; there are six hexagons containing three CheA monomers each, surrounding one that lacks the histidine kinase (Briegel *et al*., 2012; Liu *et al*., 2012; Cassidy *et al*., 2015). This CheA free hexagon can contain additional CheW.

The arrangement of receptors in highly ordered arrays is not unique to *E. coli*, but has been found in all imaged chemotactic bacteria and archaea so far (Briegel *et al*., 2009; 2015).

While we have a thorough understanding of the chemosensory system in *E. coli*, we are just beginning to unravel structure and function of such systems in other organisms. In this review, we will summarize the current knowledge about the complex chemosensory systems in *V. cholerae*, a key model system as a human pathogen with a complex life cycle. Specifically, we will highlight similarities and differences to the model system in *E. coli*, and outline some of the remaining open questions.

## 3 THE COMPLEX CHEMOSENSORY SYSTEM OF *V. cholerae*

**FIGURE 2:**
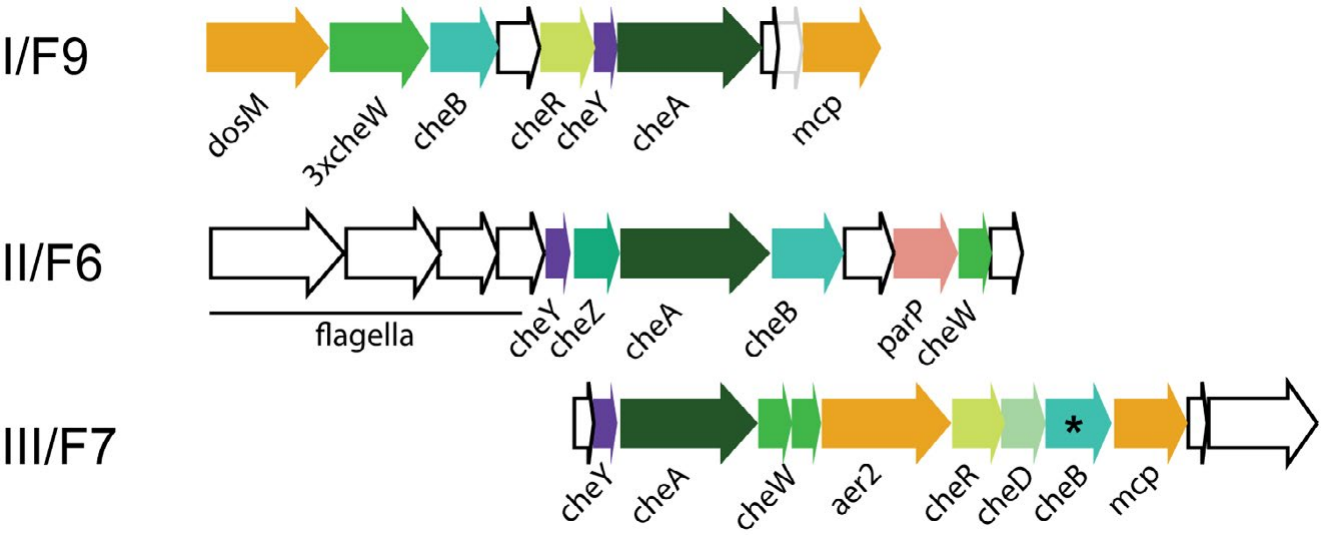
Gene clusters of *V. cholerae* encoding the core components (colored) of the three chemosensory systems. In this particular strain of *V. cholerae* O1 biovar El Tor str. N16961, F7‐CheB has an authentic frame shift (*). Other strains of *V. cholerae* have a working gene at this position

*V. cholerae's* three chemosensory operons have traditionally been named based on their location in the genome: cluster I and II are located on chromosome I, and cluster III on chromosome II (Gosink *et al*., 2002). However, this nomenclature becomes confusing when we start analyzing and comparing chemosensory systems across species. Conveniently, the landmark paper from the Zhulin group provides a phylogenomic classification of prokaryotic chemosensory systems into 19 different classes (Wuichet and Zhulin, 2010): one type IV pilus system (Tfp), one “alternative cellular functions” system (Acf), and 17 flagellar (F) systems. According to this classification scheme, *V. cholerae* cluster I belongs to the class of the F9 systems, cluster II to the F6, and cluster III to the F7 systems (Figure 2). Despite the fact that all Vibrio clusters fall under the “F” classification, the cellular role of the systems F7 and F9 remains unclear. Only the system F6 (cluster II) has been demonstrated to control flagellar motility (Gosink *et al*., 2002).

*V. cholerae* is motile by means of a single polar flagellum. The counter‐clockwise rotation of the flagellar motor pushes the cell forward, while a clockwise (cw) rotation pulls the cells backward. The switch from cw to ccw rotation of the motor is followed by a so‐called “flick” of the flagellum, which allows the cell to randomly reorient itself (Son *et al*., 2013). This run‐reverse‐flick behavior is essentially comparable to the run and tumble phases of the multi‐flagellated *E. coli*, and both swimming behaviors are controlled by chemotaxis and result in a biased random walk of the cells (Xie *et al*., 2011).

## 4 ULTRASTRUCTURE OF CHEMOSENSORY SYSTEMS IN *V. cholerae*

**FIGURE 3:**
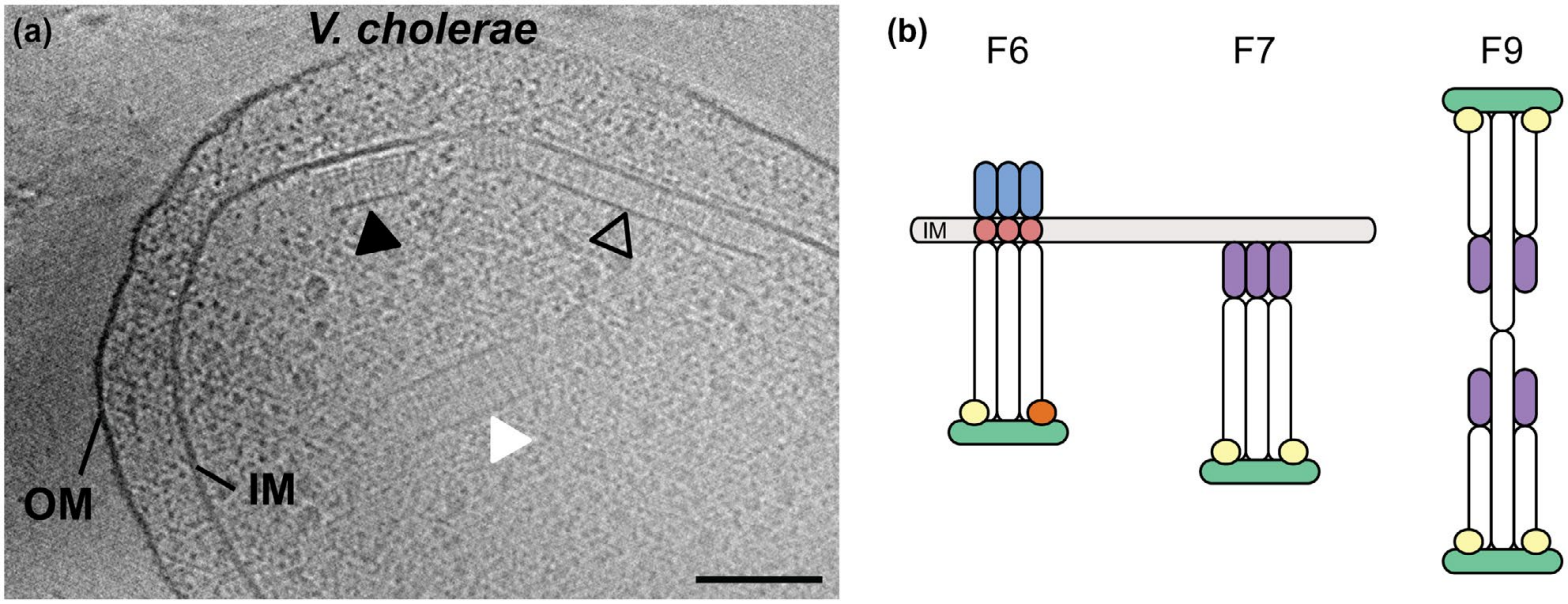
Ultrastructure of chemosensory systems in the *V. cholerae* cell. (a) The systems are marked with arrows: I/F9 (white), II/F6 (empty), and III/F7 (black). The inner membrane (IM) and outer membrane (OM) are also labeled. Scale bar: 50 nm. (b) Schematic of the three chemosensory arrays in *V. cholerae*. In all arrays (F6, F7, and F9), the cytoplasmic domains of the chemoreceptors (white) interact with the histidine kinase (green) and the scaffold proteins CheW (light yellow) and/or CheV (orange). The receptors themselves differ between the three systems: the chemoreceptors of the F6 system contain a periplasmic sensing domain (blue) and a transmembrane region (red). In contrast, the receptors of the F7 system lack any extracellular and transmembrane components and instead contain a cytoplasmic ligand input domain (magenta). The F9 system consists of two opposing receptor layers stabilized by the DosM receptor that spans between the two CheA/CheW layers. In addition, cryo‐EM revealed that at least one other cytoplasmic receptor is also part of the F9 system

All three chemosensory systems in *V. cholerae* form structurally distinct arrays in the cells (Figure 3). While the cluster II/F6 system has been observed under all tested growth conditions so far, cluster I/F9 and cluster III/F7 have so far only been observed under starvation conditions in late stationary phase (Kan *et al*., 2004; Hiremath *et al*., 2015; Ringgaard *et al*., 2015; Briegel *et al*., 2016; Ortega *et al*., 2020). The cluster II/F6 arrays are termed “short membrane arrays” (SMA). They form large clusters at the cell poles in close proximity to the single flagellum of the cells. Their physical height, measured from the inner membrane to the baseplate composed of CheA and scaffolding proteins such as CheW, is 25 nm (Briegel *et al*., 2009). They can be easily distinguished from the Cluster III/F7 “long membrane array” (LMA) that are 38 nm in height (Ortega *et al*., 2020). Additionally, the SMA have visible periplasmic domains, while the LMA do not. In contrast, cluster I/F9 arrays are purely cytoplasmic (CA) and have a characteristic double‐layered appearance with a height of 35 nm (Briegel *et al*., 2016) (Figure 3). A large collection of *V. cholerae* tomograms showing these arrays is available in the ETDB‐Caltech (Ortega *et al*., 2019).

## 5 CLUSTER I/F9 SYSTEM

The cluster I/F9 chemosensory system is functionally still poorly understood. Deletion of the CheA gene of the F9 system does not result in a detectable phenotype under standard conditions (Gosink *et al*., 2002; Hyakutake *et al*., 2005; Butler *et al*., 2006). Several studies report changes of the expression levels of cluster I proteins upon infection, see for example (Merrell *et al*., 2002; Hang *et al*., 2003). However, it should be noted that one of the commonly used lab strains, a variant of C6706, has been shown to be quorum sensing impaired (Stutzmann and Blokesch, 2016). This may affect interpretation on expression patterns in studies using this affected strain and its derivatives. The cluster formation was shown by fluorescent light microscopy to be induced under low oxygen conditions (Hiremath *et al*., 2015), suggesting it may be involved in cellular processes in microaerobic enviroments such as the intestine.

While the function of this cluster is still elusive, we have recently gained insight into the unique structure of this array. In contrast to the other two clusters, the F9 system is purely cytoplasmic. Such cytoplasmic clusters have been previously observed with cryo‐electron tomography in both bacteria (*Rhodobacter spharoides*, *V. cholerae*) (Briegel *et al*., 2014) and arachea (*Methanoregula formicia*) (Briegel *et al*., 2015).

In cluster I/F9 arrays, like all other studied prokaryotic chemorceptor arrays so far, the receptors are packed in trimers of receptor dimers that arrange in extended hexagonal arrays. In contrast to membrane‐bound arrays, these cytoplasmic clusters are formed by two oposing receptor array layers: Two base plates, formed by the kinase CheA (VC1397) and a concatenated triple CheW (VC1402) (Table S1), sandwich the receptors in between (Briegel *et al*., 2014; 2016). Deletion mutants revealed that the CheA is not essential for formation of this cluster, but reduced the number of observable arrays (Briegel *et al*., 2016). The chemoreceptor that appears to be essential for the formation of this cluster is called DosM (VC1403). This receptor has an unusual architecture: It contains two signaling domains (Table S1). This type of receptor is not unique to *V. cholerae*, but can be found in the genomes of other bacteria that possess an F9 class of chemosensory systems (Wuichet and Zhulin, 2010).

**FIGURE 4:**
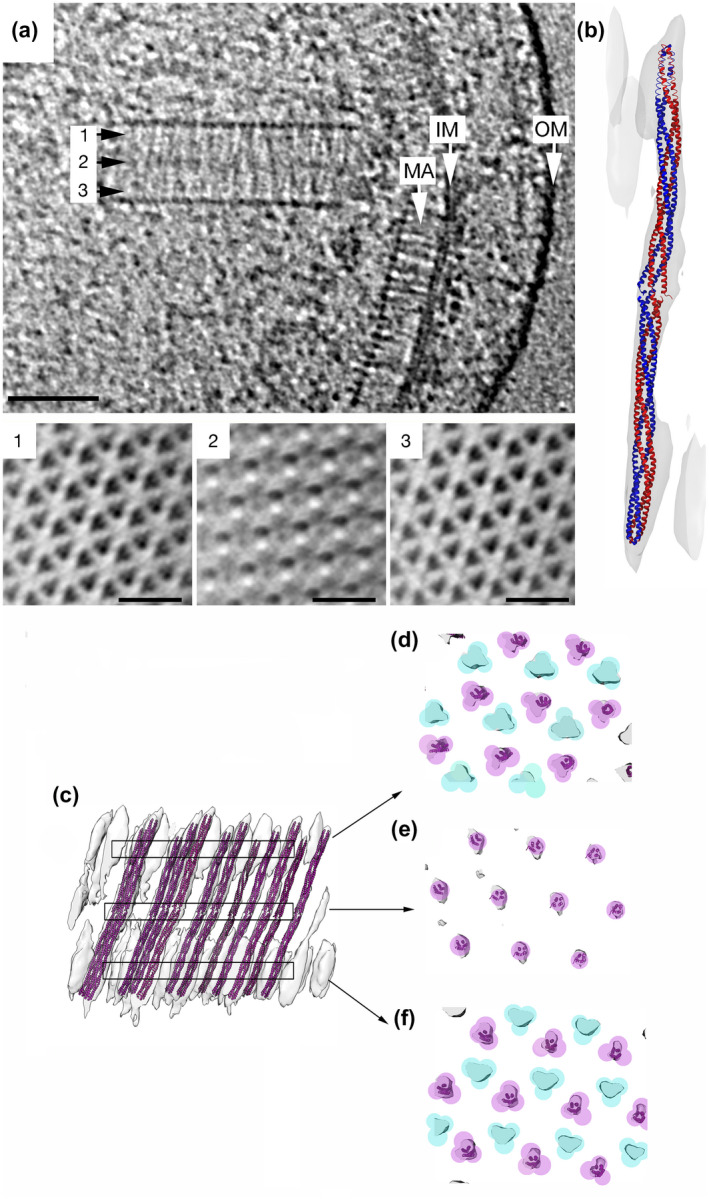
Ultrastructure of the I/F9 chemosensory system in *V. cholerae*. (a) Side view of I/F9 and II/F6 chemosensory systems and cross‐section of the I/F9 array at different heights. (b) Homology model of DosM chemoreceptor fitted in the electron density of a subtomogram average using molecular dynamics flexible fitting. (c) Side‐view of the subtomogram average of the I/F9 with multiple DosM fitted into the EM map spanning between the two CheA/CheW layers. Top view of the (c) panel at different heights: (d) close to the top CheA/CheW layer, (e) middle of the array, and (f) close to the bottom of the bottom CheA/CheW layer. Figure adapted from (Briegel *et al*., 2016)

Cryo‐electron tomography data (cryoET) revealed that this receptor arranges as an extended rod with one signaling domain at each end (Figure 4). This allows the receptor to integrate into both baseplates and act as a structural scaffold for this array (Briegel *et al*., 2016). DosM is part of every other trimer‐of‐receptor‐dimer, implying that at least one other cytoplasmic receptor is necessary for array formation. Possible candidates are VC0098, VC1406, VCA0864, and VCA1092. Which of these receptors, if not all of them, can integrate into the arrays of the F9 system remains to be determined.

## 6 CLUSTER II/F6 SYSTEM

The Cluster II/F6 system is the only one that has been shown to affect chemotactic behavior under any tested conditions so far. This was first determined by the analysis of deletion mutants of the three CheA kinases on motility swarm assays in LB soft agar (Gosink *et al*., 2002). This cluster has been implied to play a role in infection in the suckling mouse model, where it regulates colonization to the proximal, but not distal, small intestine (Millet *et al*., 2014). Interestingly, non‐chemotactic mutants were shown to also colonize the upper small intestine and exhibit heightened infectivity (Butler and Camilli, 2005). Motility per se, at least in the El Tor biotype, appears to play a role in order to penetrate the mucus layer in animal models.

Cluster II forms large short polar arrays in the vicinity of the flagellar motor (Briegel *et al*., 2009; 2016) (Figure 3). At first glance, they appear similar to the arrays of *E. coli*: the receptors are inserted into the membrane, and the periplasmic domains are easily visible in the side views of the arrays imaged by cryoET. In top view, the receptors also arrange into trimers of dimers that together form extended hexagonal arrays (Briegel *et al*., 2009).

### 6.1 Polar positioning

The polar positioning of the arrays in *E. coli* is thought to occur through a stochastic process, where new receptors insert into the membrane and diffuse freely until they ultimately form or join existing arrays at the pole (Thiem and Sourjik, 2008). The Tol–Pal complex further appears to restrict array mobility and ensures polar array localization (Santos *et al*., 2014). The polar positioning of the cluster II arrays in *V. cholerae* is accomplished through a different mechanism. Here, the arrays are anchored to the pole through the ParP/ParC system (a recent review on this topic can be found here (Ringgaard *et al*., 2018)). The protein ParP integrates into the baseplate of the chemoreceptor arrays, and binds both the chemoreceptors as well as CheA. ParP has two domains: a C‐terminal Array Integration and Formation (AIF) domain, and a ParC‐interaction domain. The AIF domain binds to the receptors as well as the LID domain of CheA. This Location and Inheritance Domain is an extra domain located between the response‐regulator docking domain P2 and the dimerization domain P3. It only occurs in species that also encode the proteins ParC and ParP in their genomes (Ringgaard *et al*., 2014). The C‐terminal domain of ParP binds to the ParA‐like ATPase ParC, which is responsible for polar anchoring of the arrays depending on the polar determinant HubP. HubP localization is dependent on the cell cycle: in newly divided cells it is localized at the old cell pole (Galli *et al*., 2017). Over time, it will start accumulating at the new pole as well. This allows for the assembly of another chemoreceptor array prior to cell division, and ensures both daughter cells start out with a functional chemotaxis system (Ringgaard *et al*., 2018). Since ParC and HubP are not interacting directly, there must be a yet‐unidentified factor linking the two proteins (Yamaichi *et al*., 2012).

### 6.2 Base plate composition and variability

ParP is not the only extra component that integrates into the baseplate of the Cluster II/F6 arrays. The genome of *V. cholerae* also encodes four fusion proteins between CheW and CheY, the so‐called CheV proteins (VC1602, CheV1; VC2006, CheV2; VC2202, CheV3; and VCA0954, CheV4), Table S1. These proteins integrate into the baseplate similar to CheW (Szurmant and Ordal, 2004). All CheV proteins are expressed together with the other cluster II proteins (Yang *et al*., 2018). In wild‐type cells, only the fluorescently tagged CheV2 was found to colocalize with the arrays.

An earlier study using swarm assays and fluorescence microscopy discovered that this CheV plays a role in chemotaxis (Hiremath *et al*., 2013).

(It is noteworthy that in this specific study cheV genes are numbered from a silent 0 instead of explicit 1: CheV(VC1602), CheV1(VC2006), CheV2(VC2202), and CheV3(VCA0954). So the labeling “CheV” used in the study (Hiremath *et al*., 2013) actually refers to the same protein “CheV1” of the later study (Yang *et al*., 2018).

However, in the absence of CheA, CheV4 also appears to integrate into the array (Yang *et al*., 2018). This illustrates that the *V. cholerae* baseplate is a variable structure and its composition likely depends on the environmental conditions and/or the life cycle stage the cells are currently exposed to.

If and under what circumstances the other two CheV proteins, CheV1 and CheV3, integrate into this array is unclear at the moment. The function of CheV is thought to assist the integration of certain receptors into the array and modulate their function (Ortega and Zhulin, 2016).

An LC‐MS proteomics analysis of the baseplate stoichiometry of *V. cholerae* under standard growth conditions revealed the following composition (Yang *et al*., 2018):

50 CheW: 7.5 CheA: 1.4 ParP: 3.8 CheV1: 1 CheV2: 4.3 CheV3: 4.9 CheV4

This indicates that the majority of the baseplate is made‐up by CheW, and the abundance of CheA is significantly less compared to *E coli*. Here, the stoichiometry is 1 CheA: 1 CheW (or 2 if the CheA less hexagons are filled with CheW instead) (Briegel *et al*., 2012; Liu *et al*., 2012; Briegel *et al*., 2014). CheA is believed to act as a structural staple, linking neighboring hexagons together. The low abundance of the structurally stabilizing CheA is likely the reason for the low stability of the Vibrio arrays. While *E. coli* arrays are known to be ultrastable (Erbse and Falke, 2009) and are structurally not affected by lysis (Briegel and Jensen, 2017), the *V. cholerae* arrays readily disassemble (Yang *et al*., 2018).

## 7 CLUSTER III/F7 SYSTEM

Like the cluster I/F9 system, the chemosensory role of cluster III/F7 in Vibrio is poorly understood. RpoS and quorum sensing have been implicated to control expression of this system both in vitro and in vivo (Ringgaard *et al*., 2015).This paper implicates that carbon starvation in an RpoS dependent, but CqsA independent manner induces the cluster III/F7 system. However, this study was performed using the C6706 strain that may have a compromised quorum sensing ability through a mutation in the regulatory protein LuxO, rendering it constitutively active (Stutzmann and Blokesch, 2016). Therefore, the quorum sensing involvement of Cluster III protein expression remains somewhat unclear.

The cluster is also polarly localized, but this localization appears to be independent of Cluster I and II (Ringgaard *et al*., 2015) The Cluster III arrays are visible in ~35% of cryoET data sets of late stationary phase cells of strain C6706 (Ortega *et al*., 2020). Deletion of either the Aer‐2 receptor (VC1092, Mlp45) or both CheW proteins and the CheA of this cluster (VCA1093, VCA1094, and VCA1095) resulted in the complete absence of visible arrays by cryoET. The receptor essential for F7 cluster formation is Aer2 (VC1092, Mlp45). This receptor is a predicted cytoplasmic receptor with a dual PAS heme domain, followed by two HAMP domains and the kinase control domain (Greer‐Phillips *et al*., 2018). This receptor was recently shown to be able to hijack the *E. coli* chemotaxis system and mediate a signaling response to oxygen (Greer‐Phillips *et al*., 2018). The cellular response upon oxygen binding to Aer2 in the context of cluster III arrays remains unknown. Despite the lack of any predicted transmembrane anchors in Aer2, the F7 arrays are clearly associated with the membrane (Ortega *et al*., 2020). The mechanism underlying this interaction with the membrane is not clear at present. CryoET data of the F7 arrays align well with the Aer2 structural model, and density layers can be seen that are corresponding to the location of the PAS domains (Ortega *et al*., 2020).

## 8 CHEMORECEPTORS IN *V. cholerae*

**FIGURE 5:**
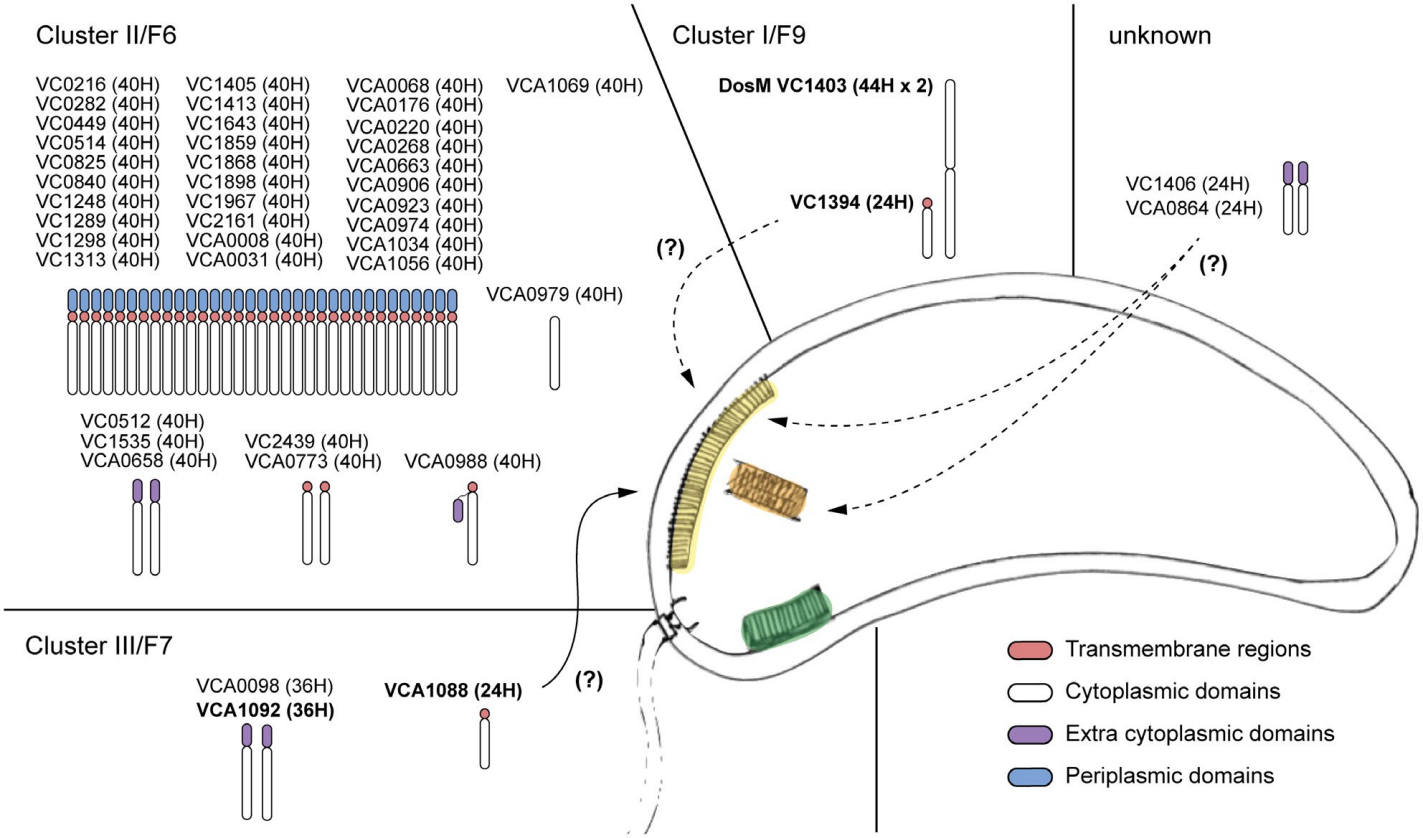
Prediction of chemoreceptors and signaling pathways in *V. cholerae*. Each chemoreceptor is represented by a simplified cartoon of its topology and labeled by their locus number and heptad classes in parentheses. Chemosensory clusters I/F9 (orange), II/F6(yellow), and III/F7 (green) are represented in the cartoon. Chemoreceptor genes encoded with core components of the system is shown in bold. The arrows show the chemoreceptors that could potentially signal through a different chemosensory pathway with some experimental evidence (solid) and with bioinformatic evidence only (dotted)

While most chemoreceptors that signal through the I/F9 and III/F7 pathways are present in the same gene cluster, there are several chemoreceptors that are predicted to signal through the II/F6 pathway. The prediction of which signaling pathway is used by a given chemoreceptor remains a tough problem in bioinformatics. However, recent work has shown that a bundle of techniques can be effective for the task (Ortega *et al*., 2017). Here, we use the MiST3 database (Gumerov *et al*., 2020) to infer pathway assignments using heptad/chemosensory class relationships and gene placement in the chromosome (Figure 5). A survey of hundreds of genomes with single chemosensory systems shows that there is a significant correlation between the heptad classes and the chemosensory classes (Ortega *et al*., 2017). For example, 36H chemoreceptors are likely to signal through F7 chemosensory systems. On the contrary, 40H chemoreceptors can signal through different classes including F6. However, so far there is no evidence that these receptors can signal through F7 systems. In addition, chemoreceptors that are encoded together with core chemosensory components have a strong possibility to be involved in the same cellular function. Using these two techniques, together with previous literature, we were able to predict the signaling pathway of all but four of the 45 chemoreceptors in *V. cholerae*. All outliers are 24H chemoreceptors. It is known that 24H chemoreceptors are found in genomes of organisms with either F7 or F6 systems. There are no known genomes with only an F9 system, but it is possible that 24H receptors signal through F9 systems (Wuichet and Zhulin, 2010).

### 8.1 The 24H chemoreceptors outliers

Two of the 24H receptors are encoded together with core chemosensory components: The receptor VC1394 is encoded in the I/F9 and the receptor VCA1088, Mlp44 encoded with III/F7. Little is known about the VC1394, however, deletion of the chemoreceptor (VCA1088, Mlp44) had no effect on the presence or abundance of the III/F7 system arrays. VCA1088 is a McpA‐like receptor: McpA‐like chemoreceptors are only found in genomes that encode an F7 system, such as in Pseudomonas‐like and Vibrio‐like systems, and always neighboring the F7 chemosensory gene cluster (Ortega *et al*., 2020). There are currently no experimental reports on the McpA‐like receptor VCA1088 available. More information is available about the McpA‐like receptor in *Pseudomonas aeruginosa*. Also known as CttP, this chemoreceptor has been involved in positive chemotaxis to trichloroethylene (Kim *et al*., 2006). Furthermore, there is experimental data showing that this receptor physically clusters together with other genes from the F6 chemosensory system controlling flagellar motility (Güvener *et al*., 2006). This implies that the role of McpA‐like receptors may be related to the biological function of the F7 chemosensory systems and suggest a possible functional link between the F6 and F7 systems. Like the McpA receptor, the VC1394 present in the F9 system has the same topology: a 24H signaling domain and a transmembrane region, Table S1. It is possible that VC1394 also integrates into the II/F6 arrays rather than those of the I/F9 system.

The other two outliers, VC1406 and VCA0864, both have two PAS domains instead of transmembrane regions, Table S1. This implies they may sense intracellular oxygen levels or redox potential (Taylor and Zhulin, 1999). It is possible that the I/F9 system is structurally composed of DosM together with one or both of these receptors (VC1406 or VCA0864) together with, or instead of, the chemoreceptor encoded in the I/F9 gene cluster, VC1394.

### 8.2 The chemoreceptors of cluster II/F6

In order to integrate into an array, a receptor has to physically properly fit: The length between the receptor tip that interacts with the baseplate to the transmembrane region has to match the other receptors in the array. Experiments in *E. coli* showed that either extension or truncation of a receptor by one heptad alone was sufficient to prevent integration into the main cluster, and the receptor with the altered length formed its own distinct array (Herrera Seitz *et al*., 2014). Most receptors (32 of 43) in *V. cholerae* match the same criteria: They are membrane‐bound receptors of the 40H (heptad) class (Alexander and Zhulin, 2007). Furthermore, 40H is the predicted receptor class to interact with the F6 chemosensory system (Wuichet and Zhulin, 2010; Ortega *et al*., 2017). Thus, the 40H receptors with a transmembrane region are thought to be able to integrate into cluster II arrays.

The cluster II receptor composition is likely variable and dependent on environmental conditions. A recent study reported that the abundance of 40H receptors varied significantly based on a proteomics analysis of overnight cultures grown in LB compared to viable but non culturable cells, which are grown for months in various natural water microcosms (Brenzinger *et al*., 2019). The non‐ultrastability of the cluster II arrays may facilitate receptor turnover to allow the cluster to rapidly adapt to environmental conditions (Brenzinger *et al*., 2019).

### 8.3 Signals for 40H receptors

While the ligands for most of the *V. cholerae* receptors are still unknown, some have been uncovered in the past years. For example, VC2161 (also called Mlp24 or McpX) senses amino acids such as l‐arginine, L‐proline, and L‐serine (Nishiyama *et al*., 2012). Besides amino acids, Taurine (2‐aminoethanesulfonic acid, a major component of bile) is also a strong attractant for the receptor VCA0923 (Mlp37) (Nishiyama *et al*., 2016).

Another 40H receptor, VCA0658, has been shown to be involved in aerotaxis (Boin and Hase, 2007). Even though this receptor lacks a transmembrane region (Gumerov *et al*., 2020), it is highly likely that it integrates into the cytoplasmic region of the cluster II array because aberrant chemotaxis behavior is observed on swarm and swimming assays (Boin and Hase, 2007).

Other less well‐specified functions of the receptors have been reported in the past. For example, the receptor VCA0220/Mlp30/HlyB has been implicated in pathogenicity and Hemolysin secretion control (Alm and Manning, 1990; Jeffery and Koshland, 1993). Both VC0825/TcpI/Mlp7 (Harkey *et al*., 1994; Chaparro *et al*., 2010) as well as VC0840/AcfB/Mlp8 have been implicated as colonization determinants (Everiss *et al*., 1994a; 1994b).

Although the signaling capabilities of the *V. cholerae* chemotaxis system are not fully understood, we predict it senses a large variety of input signals. Several of the chemoreceptors in *V. cholerae* contain cytoplasmic PAS domains that are known to sense different small molecules, most notably oxygen (Henry and Crosson, 2011). Additionally, 31 of the 40H receptors have periplasmic sensing domains. The PFAM database classifies these periplasmic domains into different categories: as sCache_2 (5), dCache_1 (7), 4HB_MCP_1 (4), or as unclassified regions (15) that contained ~50 amino acids between the transmembrane regions with no match to the PFAM database. It is important to note that even small changes in the amino‐acid sequences of these domains may dramatically affect their signaling specificities. In summary, this large diversity of ligand binding domains likely facilitates accurate navigation in changing environments during the complex life cycle of *V. cholerae*.

## 9 CONCLUSION

The rich chemosensory system in *V. cholerae* remains a formidable target to study the complex arrangements of chemosensory systems. The similarities with other emergent model organisms, such as *P. aeruginosa*, uncovered by recent bioinformatics work, serve to bridge the gap between these systems and allow for extrapolation of hypothesis and a better understanding of the signaling system as a whole. While the functions of two out of three chemosensory systems in *V. cholerae* remain unknown, recent work has significantly advanced the field. Taken together, these recent studies allow for the generation of a complex signaling hypothesis in this organism. It supports a functional cross talk of I/F9 and III/F7 clusters with II/F6 cluster via specialized chemoreceptors.

In our opinion three intriguing questions remain at the center of *V. cholerae's* chemosensory systems studies:

1. What is the function of I/F9 and III/F7 and how are they related to the II/F6 system?
2. What is the mechanism that attaches the III/F7 system to the membrane?
3. How does the II/F6 system balance signals of so many chemoreceptors?

Finally, further experimental research will be essential to confirm our proposed prediction of matching chemoreceptors to signaling pathways. This will further improve predicting models of chemosensory signaling systems in bacteria and archaea.

## Supporting information

Table S1
