# Supplementary material for: The chemosensory systems of Vibrio cholerae
Source: Mol Microbiol. 2020 May 13;114(3):367–76. doi: 10.1111/mmi.14520 (PMC7534058; doi:10.1111/mmi.14520)
Supplement: Supplementary file 1 — Table S1 [file MMI-114-367-s001.pdf]

Table I: Chemosensory proteins of *V.cholerae*.

Cluster I/F9

Locus (old locus)	Gene name	Product	Type	Class	Domain Architecture**	Role	Assignment evidence
VC_RS06750 (VC1394)		methyl-accepting chemotaxis protein	mcp	24H	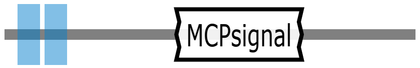	signal input	GN
VC_RS06755		*frame shift*					
VC_RS06760 (VC1396)		hypothetical protein				unknonw	GN
VC_RS06765 (VC1397)		chemotaxis protein CheA	chea	F9	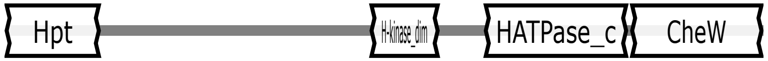	signal processing	CC
VC_RS06770 (VC1398)		response regulator	chey		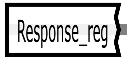	signal output	GN
VC_RS06775 (VC1399)		protein-glutamate O-methyltransferase CheR	cher	F9	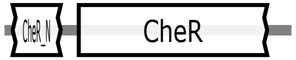	adaptation	GN, CC
VC_RS06780 (VC1400)		hypothetical protein				unknown	GN
VC_RS06785 (VC1401)		chemotaxis protein CheB	cheb	F9	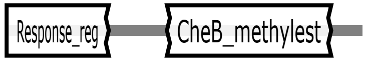	adaptation	GN, CC
VC_RS06790 (VC1402)		chemotaxis protein W	chew		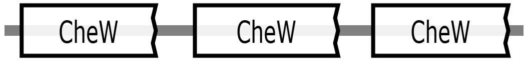	scafold	GN, (Briegel et al., 2016)

Locus (old locus)	Gene name	Product	Type	Class	Domain Architecture**	Role	Assignment evidence
VC_RS06795 (VC1403)		methyl-accepting chemotaxis protein	mcp	44H	MCPsignal – MCPsignal	signal input	GN, (Briegel et al., 2016)

Cluster II/F6

Locus (old locus)	Gene name	Product	Type	Class	Domain Architecture**	Role	Assignment evidence
VC_RS09920 (VC2059)		chemotaxis protein CheW	chew		CheW	scaffold	GN, (Butler and Camilli, 2006)
VC_RS09925 (VC2060)		chemotaxis protein CheW	chew		CheW	localization	GN, (Ringgaard et al., 2018)
VC_RS09930 (VC2061)		ParA family protein			AAA_31	localization	GN, (Ringgaard et al., 2018)
VC_RS09935 (VC2062)		chemotaxis response regulator protein-glutamate methylesterase	cheb	F6	Response_reg – CheB_methylest	adaptation	CC, GN
VC_RS09940 (VC2063)		chemotaxis protein CheA	chea	F6	Hpt – H-kinase_dim – HATPase_c – CheW	signal processing	CC

Locus (old locus)	Gene name	Product	Type	Class	Domain Architecture**	Role	Assignment evidence
VC_RS09945 (VC2064)		protein phosphatase CheZ	chez	F6	CheZ	signal termination	CC, GN
VC_RS09950 (VC2065)		response regulator	chey		Response_reg	signal output	GN

Cluster II/F6 - associated

Locus (old locus)	Gene name	Product	Type	Class	Domain Architecture**	Role	Assignment evidence
VC_RS10640 (VC2201)		protein-glutamate O-methyltransferase	cher	F6	CheR_N CheR	adaptation	CC
VC_RS10645 (VC2202)		chemotaxis protein CheV	chev	F6	CheW Response_reg	adaptation	CC
VC_RS07755 (VC1602)		chemotaxis protein CheV	chev	F6	CheW Response_reg	adaptation	CC
VC_RS09650 (VC2006)		chemotaxis protein CheV	chev	F6	CheW Response_reg	adaptation	CC
VC_RS17860 (VCA0954)		chemotaxis protein CheV	chev	F6	CheW Response_reg	adaptation	CC

Chemoreceptors away from cluster (prediction)

Locus (old locus)	Gene name	Product	Type	Class	Domain Architecture**	Role	Assignment evidence
VC_RS01060 (VC0216)		methyl-accepting chemotaxis protein	mcp	40H	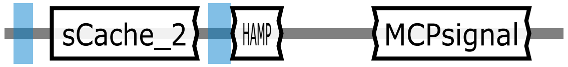	signal input	CxH
VC_RS01370 (VC0282)		methyl-accepting chemotaxis protein	mcp	40H	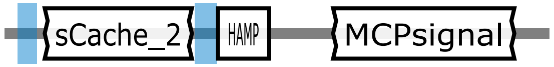	signal input	CxH
VC_RS02285 (VC0449)		methyl-accepting chemotaxis protein	mcp	40H	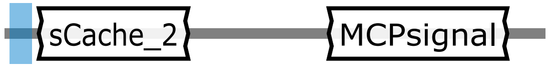	signal input	CxH
VC_RS02600 (VC0512)		methyl-accepting chemotaxis protein	mcp	40H	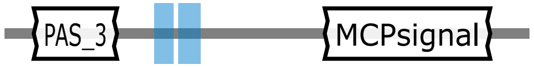	signal input	CxH
VC_RS02610 (VC0514)		methyl-accepting chemotaxis protein	mcp	40H	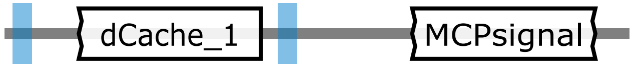	signal input	CxH
VC_RS04105 (VC0825)	*tcpI*	toxin-coregulated pilus methyl-accepting chemotaxis protein TcpI	mcp	40H	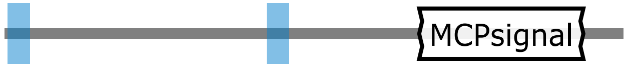	signal input	CxH
VC_RS04180 (VC0840)	*acfB*	methyl-accepting chemotaxis protein AcfB	mcp	40H	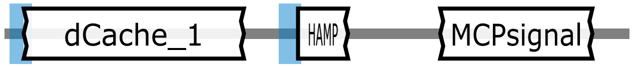	signal input	CxH
VC_RS06095 (VC1248)		methyl-accepting chemotaxis protein	mcp	40H	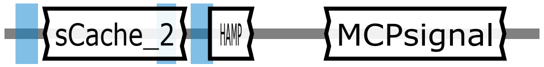	signal input	CxH
VC_RS06290 (VC1289)		methyl-accepting chemotaxis protein	mcp	40H	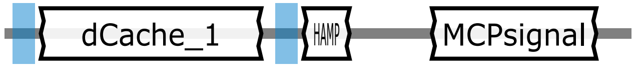	signal input	CxH
VC_RS06325 (VC1298)		methyl-accepting chemotaxis protein	mcp	40H	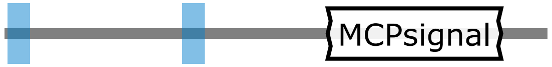	signal input	CxH

Locus (old locus)	Gene name	Product	Type	Class	Domain Architecture**	Role	Assignment evidence
VC_RS06400 (VC1313)		methyl-accepting chemotaxis protein	mcp	40H	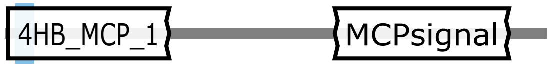	signal input	CxH
VC_RS06800 (VC1405)		methyl-accepting chemotaxis protein	mcp	40H	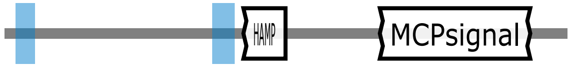	signal input	CxH
VC_RS06835 (VC1413)		methyl-accepting chemotaxis protein	mcp	40H	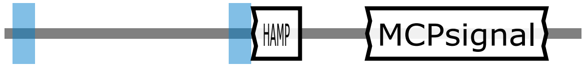	signal input	CxH
VC_RS07420 (VC1535)*		methyl-accepting chemotaxis protein	mcp	40H	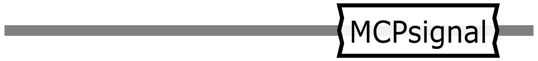	signal input	CxH
VC_RS07960 (VC1643)		methyl-accepting chemotaxis protein	mcp	40H	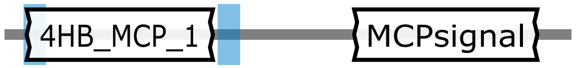	signal input	CxH
VC_RS08970 (VC1859)		methyl-accepting chemotaxis protein	mcp	40H	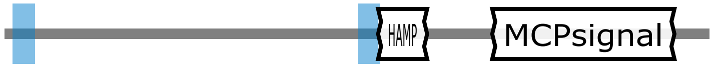	signal input	CxH
VC_RS09015 (VC1868)		methyl-accepting chemotaxis protein	mcp	40H	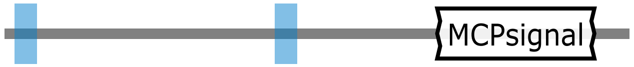	signal input	CxH
VC_RS09155 (VC1898)		methyl-accepting chemotaxis protein	mcp	40H	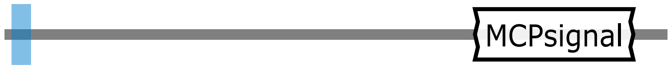	signal input	CxH
VC_RS09470 (VC1967)		methyl-accepting chemotaxis protein	mcp	40H	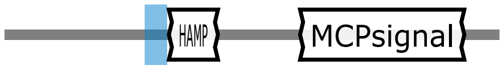	signal input	CxH
VC_RS10385 (VC2161)	*mlp24*	methyl-accepting chemotaxis protein Mlp24	mcp	40H	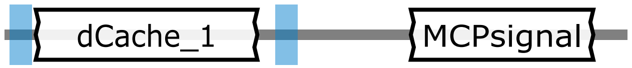	signal input	CxH, (Nishiyama *et al.*, 2012)
VC_RS11750 (VC2439)		methyl-accepting chemotaxis protein	mcp	40H	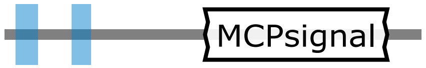	signal input	CxH

Locus (old locus)	Gene name	Product	Type	Class	Domain Architecture**	Role	Assignment evidence
VC_RS13460 (VCA0008)		methyl-accepting chemotaxis protein	mcp	40H	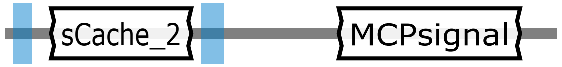	signal input	CxH
VC_RS13555 (VCA0031)		methyl-accepting chemotaxis protein	mcp	40H	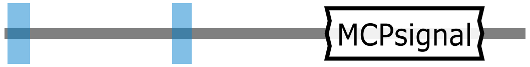	signal input	CxH
VC_RS13710 (VCA0068)		methyl-accepting chemotaxis protein	mcp	40H	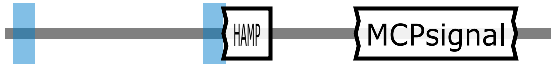	signal input	CxH
VC_RS14190 (VCA0176)		methyl-accepting chemotaxis protein	mcp	40H	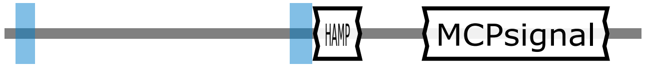	signal input	CxH
VC_RS14390 (VCA0220)	*mlp30*	methyl-accepting chemotaxis protein	mcp	40H	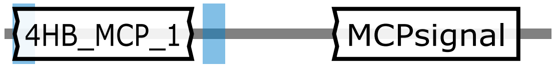	signal input	CxH
VC_RS14605 (VCA0268)		methyl-accepting chemotaxis protein	mcp	40H	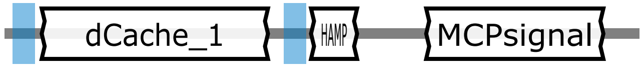	signal input	CxH
VC_RS16535 (VCA0658)		methyl-accepting chemotaxis protein	mcp	40H	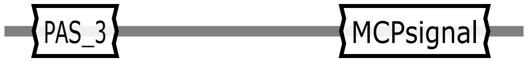	signal input	CxH
VC_RS16555 (VCA0663)		methyl-accepting chemotaxis protein	mcp	40H	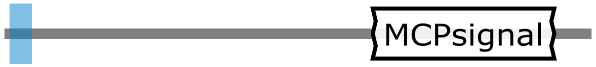	signal input	CxH
VC_RS17050 (VCA0773)		methyl-accepting chemotaxis protein	mcp	40H	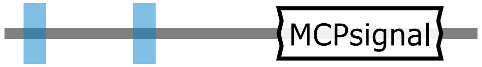	signal input	CxH
VC_RS17645 (VCA0906)		methyl-accepting chemotaxis protein	mcp	40H	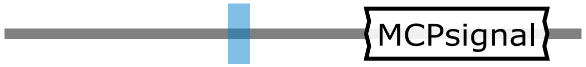	signal input	CxH
VC_RS17720 (VCA0923)	*mlp37*	methyl-accepting chemotaxis protein Mlp37	mcp	40H	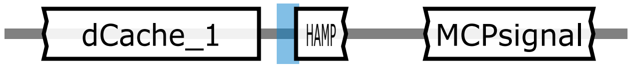	signal input	CxH, (Nishiyama *te al.*, 2016)

Locus (old locus)	Gene name	Product	Type	Class	Domain Architecture[**]	Role	Assignment evidence
VC_RS17940 (VCA0974)		methyl-accepting chemotaxis protein	mcp	40H	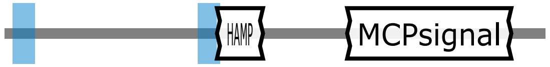	signal input	CxH
VC_RS17965 (VCA0979)[*]		methyl-accepting chemotaxis protein	mcp	40H	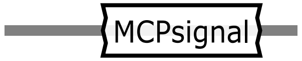	signal input	CxH
VC_RS18015 (VCA0988)		methyl-accepting chemotaxis protein	mcp	40H	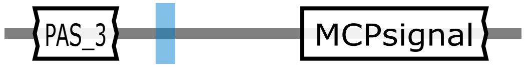	signal input	CxH
VC_RS18220 (VCA1034)		methyl-accepting chemotaxis protein	mcp	40H	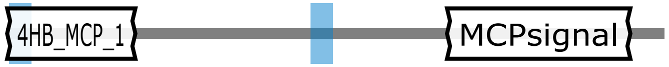	signal input	CxH
VC_RS18325 (VCA1056)		methyl-accepting chemotaxis protein	mcp	40H	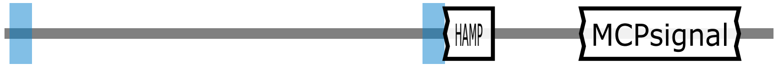	signal input	CxH
VC_RS18380 (VCA1069)		methyl-accepting chemotaxis protein	mcp	40H	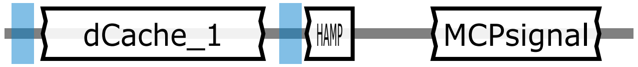	signal input	CxH

Cluster III/F7

Locus (old locus)	Gene name	Product	Type	Class	Domain Architecture[**]	Role	Assignment evidence
VC_RS18450 (VCA1085)		DUF342 domain-containing protein			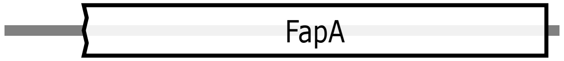	unknown	GN, (Ortega et al., 2019b)
VC_RS18455 (VCA1086)		SpoIIE family protein phosphatase			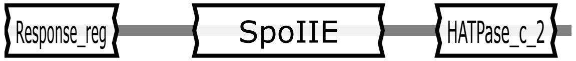	unknown	GN, (Ortega et al., 2019b)

Locus (old locus)	Gene name	Product	Type	Class	Domain Architecture**	Role	Assignment evidence
VC_RS18460 (VCA1087)		STAS domain-containing protein			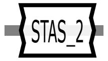	unknown	GN, (Ortega et al., 2019b)
VC_RS18465 (VCA1088)		methyl-accepting chemotaxis protein	mcp	24H	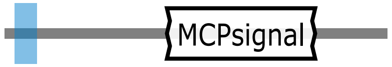	signal input	GN
VC_RS18470		frame shift					
VC_RS18475 (VCA1090)	*cheD*	chemoreceptor glutamine deamidase CheD	ched	F7	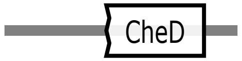	adaptation	CC, GN
VC_RS18480 (VCA1091)		protein-glutamate O-methyltransferase CheR	cher	F7	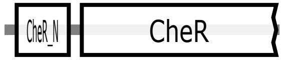	adaptation	CC, GN
VC_RS18485 (VCA1092)		HAMP domain-containing protein	mcp	36H	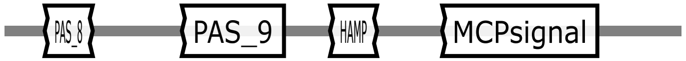	signal input	GN, CxH, (Greer-Phillips et al., 2018), (Ortega et al., 2019b)
VC_RS18490 (VCA1093)		purine-binding chemotaxis protein CheW	chew		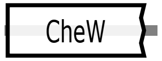	scaffold	GN
VC_RS18495 (VCA1094)		purine-binding chemotaxis protein CheW	chew		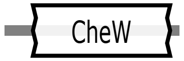	scaffold	GN

Locus (old locus)	Gene name	Product	Type	Class	Domain Architecture**	Role	Assignment evidence
VC_RS18500 (VCA1095)		chemotaxis protein CheA	chea	F7	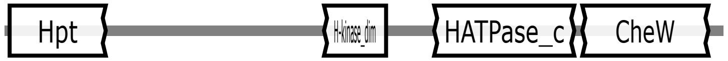	signal processing	CC, GN
VC_RS18505 (VCA1096)		response regulator	chey		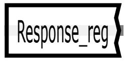	signal output	GN

Chemoreceptors away from cluster III (prediction)

Locus (old locus)	Gene name	Product	Type	Class	Domain Architecture**	Role	Assignment evidence
VC_RS00480 (VC0098)		methyl-accepting chemotaxis protein	mcp	36H	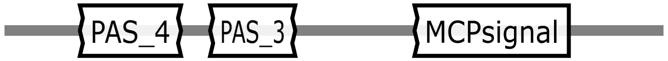	signal input	CxH

Other chemotaxis proteins

Locus (old locus)	Gene name	Product	Type	Class	Domain Architecture**	Role	Assignment evidence
VC_RS01890 (VC0377)		chemotaxis protein CheX	checx		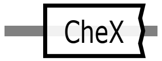	adaptation	
VC_RS05320 (VC1083)		chemotaxis protein CheC	checx		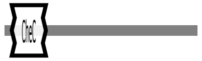	adaptation	
VC_RS05860 (VC1197)		DUF3334 family protein	other		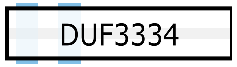	unknown	

Other chemoreceptors

Locus (old locus)	Gene name	Product	Type	Class	Domain Architecture**	Role	Assignment evidence

Locus (old locus)	Gene name	Product	Type	Class	Domain Architecture**	Role	Assignment evidence
VC_RS06805 (VC1406)		PAS domain S-box protein	mcp	24H	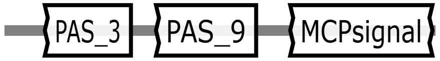	signal input	
VC_RS17440 (VCA0864)		PAS domain S-box protein	mcp	24H	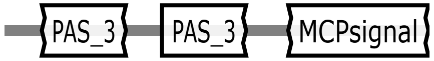	signal input	

* Protein found in newer version of the genome.

** Domain architecture prediction was taken from MiST3(Gumerov *et al*., 2020). Transmembrane regions are marked in blue.

Abreviations: CC - Chemotaxis classifiction with HMM models, CxH - Chemotaxis class vs Heptad class, GN - Gene neighborhood.
